# Organoids: fundamentals, present and future

**DOI:** 10.17843/rpmesp.2022.392.10203

**Published:** 2022-06-30

**Authors:** José Amiel-Pérez, José Amiel-Sáenz, María Amiel-Torrelio

**Affiliations:** 1 Instituto de Medicina Regenerativa, Universidad Científica del Sur, Lima, Peru. Universidad Científica del Sur Instituto de Medicina Regenerativa Universidad Científica del Sur Lima Peru; 2 Georgia Southern University Statesboro, Georgia, Estados Unidos. Georgia Southern University Georgia Southern University Statesboro Georgia USA

**Keywords:** Induced pluripotent stem cells, Gastrulation, Design matrix, Three-dimensional cultures, Cell differentiation, Signal transduction, Cell maturation, Microchip, Embryonal stem cells, Growth factors

## Abstract

Organoids are tiny structures, mainly generated from induced pluripotent stem cells, which are cultured in the laboratory while retaining their innate or acquired characteristics. They have the potential to reproduce biological development processes, model pathological processes that will enable the discovery of new drugs and promote regenerative medicine. However, these processes require constant improvement because variations may have occurred in the constitution of the organs. Therefore, this article aims to review updated information on organoids and their basic and recent experimental processes, starting with gastrulation, in an attempt to mimic, as much as possible, the formation of the three layers: ectoderm, mesoderm and endoderm; as well as the information regarding the factors involved in the induction, differentiation and maturation during the generation of organoids. Likewise, the design and preparation of highly specialized culture media that allow obtaining the selected organ with the highest precision and safety. We searched for original and review articles published in PubMed, Nature and Science. Articles were selected for their abstracts and full text. The conclusions of this article highlight the future advantages in the use and applications of organoids.

## INTRODUCTION

Organoids are miniaturized cellular structures derived from primary tissues or differentiated stem cells that are self-organized three-dimensionally (3D) *in vitro* and show physical architecture and organic functionality similar to those of the target organs: brain, liver, kidneys, stomach, intestines, and many more, allowing the modeling of both normal and sick organs ^1^ ^-^ ^5^; and have the potential to reproduce biological developmental processes ^6^ ^,^ ^7^. These organoids will help to model pathological processes and reveal unknown aspects that will allow the discovery of new drugs, verify the quality of drugs, perform toxicological tests ^8^ ^-^ ^10^ as well as more precise medical treatments conducive to personalized and regenerative medicine. The construction of organoids that can functionally replace (even partially) the function of a damaged organ is projected as a strategy of regenerative medicine ^11^. The aim of this article is to review the updated information on organoids and their basic and recent experimental processes.

## SEARCH AND SELECTION STRATEGY

We searched for original and review articles published in PubMed, Nature and Science. The search was limited to articles in English and Spanish. The words: organoids, gastrulation, cell culture, designer matrices, signaling pathways, microchips and COVID-19 were used. The articles were identified by titles, approximately 250-300, only those referring to the chosen topics. The abstracts of 102 articles were selected, and after an evaluation of the full text, 42 were selected and are shown in this article and subsequently expanded to 70 by including specific applications of the techniques to obtain organoids corresponding to various organs: brain, intestines, kidneys and applications for COVID-19. The remaining 28 references are available in the supplementary material.

## SELF-ORGANIZATION

In 1907, Wilson published his experimental paper “A new method by which sponges may be artificially reared”, which showed how sponge cells divided into individual cells and separated from each other had the ability to self-organize, thus reconstituting the living organism ^12^.

Subsequently, some articles were published about experiences in which the complete reconstitution of organs by cell reaggregation was obtained *in vitro*. However, the most notable advances in the area of organoid development have been achieved with the improvement of three-dimensional cultures and techniques derived from the use of induced pluripotent stem cells.

## CELL CULTURES

Stem cells reproduced in two-dimensional layers grow on flat surfaces and do not represent the original cells, they lose their phenotype and are not suitable for generating organoids, because they do not show cell-cell or cell-matrix interactions or mimic cell functions and signaling pathways. In contrast, three-dimensional (3D) cultures exhibit a more accurate representation of the organism’s natural environment. Therefore, they are very important for organoid formation.

The medium in which they are cultured is important for the growth, development and subsequent differentiation of stem cells. In *in vivo *systems, the concepts of microenvironment and niche (constituted by the outer layers of the cells immediately adjacent to the microenvironment) are important, especially the extracellular matrix and the set of specific interactions between the cells in that region. The use of matrices that mimic this interaction is key for *in vitro* systems, in which organoids are to be generated.

The generation of organoids requires primary tissues as a primary component, which are those that make up the animal and human organism, or stem cells, in particular induced pluripotent stem cells (iPSC). These iPSCs are the most appropriate for derivation into organoids and do not have ethical impediments ^13^, as do embryonic stem cells (ESCs), which prevent the development of the embryo, making human life impossible.

## INDUCED PLURIPOTENT STEM CELLS

Induced pluripotent stem cells are self-renewing ^14^ and can provide the physical and biochemical signals needed to create a human organoid. The physical signals are fundamental to constitute the morphological architecture of the organoid, providing support for cell-cell connection and cell growth; the components of the culture media, most notably collagen and laminin are also involved in this task. Biochemical signals are responsible for differentiation, proliferation and self-renewal in the process of organoid generation, complemented by Wnt (Wingless and Int-1), gastrin, retinoic acid, HDAC inhibitors, EGF. They are required to emulate the cellular microenvironment, to understand it better, its microbiota and, particularly, its vascularization ^15^ ^,^ ^16^.

A few years ago, adult stem cells were identified from bone marrow and fat tissue, these derive into cells from more than one germ layer ^17^. In 1998, Thomson *et al*. ^18^ cultured *in vitro* pluripotent cells taken from the blastocyst. They used enrichment culture media that stimulated their growth. Thus, the cultured stem cells again change their asymmetric characteristic and become symmetric stem cells by generating two equal cells that multiply and expand rapidly. The study by Thomson *et al*. was pioneering in obtaining stem cells from the inner mass of the blastocyst and is very important because it was an experience that precedes Yamanaka’s later studies ^19^. In 2007, Takahashi *et al*. ^20^ succeeded in obtaining human induced pluripotent stem cells by the same method.

Because of the versatility of these cells, scientists used them to obtain healthy or sick organoids, preserving their mutated or unaltered genetic traits. To achieve this, the cells to be processed had to be previously transformed into induced pluripotent stem cells; it was then necessary to take the appropriate samples and apply the updated techniques proposed by Yamanaka. Once induced, they should be expanded and differentiated to recapitulate the selected organ.

## GASTRULATION

Gastrulation is a process that takes place after the formation of the blastula and forms the embryonic disc as a consequence of the migration of cell populations that are directed to the epiblast originating the formation of the three embryonic layers.

During the natural development of the embryo, and after the formation of the morula, the blastocyst is formed, a transient embryonic structure that appears between 4-6 days after fertilization, prior to its implantation in the endometrium where 70-100 cells called blastocysts are formed, which form the inner cell mass (ICM). The cells from the ICM are pluripotent and are found inside the central cavity or blastocele, forming the trophectoderm (TE) that will constitute the placenta and the amniotic membranes.

Gastrulation begins at the end of the second week, this process takes place after the formation of the blastula and forms the embryonic disc as a consequence of the migration of cell populations that go to the epiblast, originating the formation of the three embryonic layers: ectoderm, mesoderm and endoderm, a process that leads to the cellular specifications for the natural generation of the different organs. It ends at the end of the fourth week.

The cells of the embryonic disc, consisting of a flattened mass, are organized in two layers, one that will be part of the dorsal region of the future embryo, called epiblast, and the other, the hypoblast, in the lower part. Both layers form the blastoderm, which is the origin of all embryonic cells and tissues and also of part of the extraembryonic ones.

The formation of new organisms requires the creation of various laminae capable of forming all the constituent elements of the three embryonic layers: ectoderm, mesoderm and endoderm ^21^. Initially, these layers are only two, so the formation of a third layer, the mesoderm, becomes imperative, which is then added to the previous two, epiblast and hypoblast, provided by the lamellar embryonic disc, the trilaminar embryo ^22^.

To this effect, some epiblast cells migrate, a part of them will be introduced between the epiblast and hypoblast layers towards the blastocele and the other will be incorporated into the group of cells that constitute the hypoblast layer. This migration results in the formation of the primitive line, which is the morphological aspect that the cells present when they are entering through it to form the mesoderm; this migratory process is called invagination and generates a structure of elongated aspect called notochord, which will later induce the formation of the neural tube, the neural plate and the vertebral bodies ^23^.

Invagination causes a decrease in the size of the blastocoelic cavity, specified by fibroblast growth factor-8 (FGF-8), which decreases the concentration of E-cadherin, protein that holds together the epiblast cells, thus facilitating cell migration. This decrease allows the formation of a new cavity, the gastrocoel, future intestine.

The epiblast cells that did not migrate and remained will constitute the ectoderm; the layer that has just resulted from the invagination is the mesoderm, and the new one constituted by the set of hypoblast cells plus the epiblast cells, is the endoderm.

López *et al*. ^24^ emphasize that the three embryonic laminae develop simultaneously and form different organs with their own specific characteristics for each one, with important molecular, cellular and tissue interactions among them. They also mention that many of the data obtained in embryology and the most relevant in the processes of gastrulation come from the study of birds (hens) but that, undoubtedly, they are transferable to the human species. The development of each layer is summarized below.

### Ectoderm

It constitutes the outer layer of the embryo and is part of the amniotic sac that surrounds the embryo and contains the amniotic fluid. The central part, the neural ectoderm, will form the nervous system and the non-neural ectoderm will be the epidermis.

### Mesoderm

It forms a large number of organs and is the intermediate layer resulting from the internalization of the blastula of the upper layer cells. The notochord, its most important element, has a fundamental role in the processes of neural induction. It has been shown that differentiation of the neural ectoderm does not occur when the notochord is removed. The mesoderm itself is divided into three sectors: the paraxial, intermediate and lateral mesoderm. The paraxial mesoderm forms the pairs of somites. The cells that compose the somites differentiate into three types of cellular structures: myotome, dermatome and sclerotome.

### Endoderm

**Figure 1:**
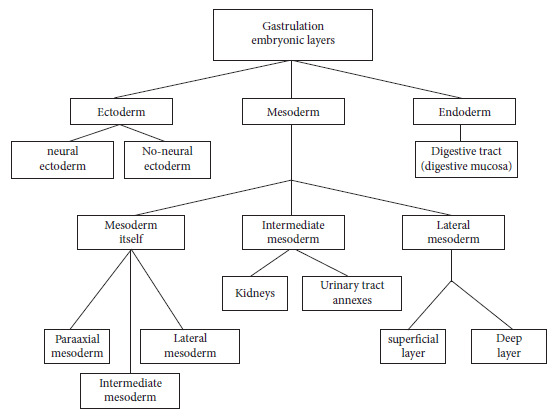
Formation of the three embryonic layers in the natural process of gastrulation

It is the inner embryonic layer, which with minimal changes follows the embryonic incurvation process from the mouth to the anus, forming the digestive tract and particularly the digestive mucosa (Figure 1).

The cardiovascular and nervous systems are formed during the third week of gestation ^25^. The circulatory system is the first unit to function and the heart does so as soon as it is required to meet the needs of oxygen and nutrients ^26^.

## DIFFERENTIATION IN NATURE

Specific gene expression is responsible for the coding and production of proteins that signal and transmit intercellular information and are the generators of intercellular information, a mechanism in which inducing and repressor molecules and control procedures participate. This gene regulation involves transcription factors that modulate the expression of certain genes and signaling pathways that, using a signaling procedure, establish intercellular communication by which a molecule called ligand binds to the surface of a cell receptor that transmits and activates, through a molecule or a chain of molecules (signaling pathway), the intracellular processes of cellular, tissue and organoid formation. For example, the role of the BMP4 ligand, which is the bone morphogenetic protein encoded by the *Bmp4* gene, is important for neurodifferentiation. When there is no *Bmp4* activity, the dorsal ectoderm forms neural tissue by default; then, the notochord produces the anti-BMP compounds noggin, follistatin and chordin, inhibitors of the protein. Molecular interactions stimulate ectodermal cells for transformation to neural tissue. The subdivision of the central nervous system is regulated by the *Otx2*, *Gbx2* and *Hox* genes that regulate regional distribution. The expression of the *Otx2* gene is important for the development of the prosencephalon and midbrain regions. The *Gbx2* and *Hox* genes could be involved in the generation of the rhombencephalon. The regionalization of the neural tube is evident in the hindbrain region with the *Hox* gene.

The first expression corresponds to the evolution and differentiation of the heart ^27^ ^,^ ^28^ and is initiated by the transcription factor Nkx-2.5, resulting from the induction of the fibroblast growth factors (FGFs) and the bone morphogenetic protein (BMP) ^29^, these are bone morphogenetic proteins that are expressed in the endoderm located next to the precardiac mesoderm and engage these cells as pathways of cardiac differentiation ^30^. Table 1 shows the main cell induction and differentiation factors in different tissues.

**Table 1:** Induction, differentiation and maturation factors for organoid generation.

Organoids	Differentiation elements
Brain	N2 supplement, NEAA, heparin for neural induction. Insulin, 2-mercaptoethanol for differentiation. Vitamin A, retinoic acid for maturation.
Intestines	Activin-A, BMP4, Wnt/beta-catenin signaling, Lgr5 marker for endoderm induction. FGF4, Wnt3A ligand. Respondin-1 for differentiation. Gremlin and Noggin, EGF, FGF4, Wnt, afamin, ZRNF3, RFN43 (ubiquitinases) for maturation.
Liver	Activin-A for endoderm induction. BMP4, FGF2, hepatocyte growth factor for differentiation. Oncostatin-M for maturation.
Kidneys	Wnt, GSK3α inhibitor for induction of intermediate mesoderm. FGF9 for differentiation
Lungs	Activin-A for endoderm induction. Wnt, BMP, FGF, cAMP. Y-glucocorticoids for differentiation.

Activin-A: dimeric structure protein; afamin: glycoprotein that transports vitamin E; BMP: bone morphogenetic protein; BMP4: bone morphogenetic protein 4 is a protein-coding gene; cAMP: cyclic adenosine monophosphate, second messenger used for the induction of intracellular signals; EGF: epidermal growth factor is a protein that stimulates cell growth and differentiation by combining with its receptor EGFR; FGF: fibroblast growth factor-23 is a protein synthesized in the osteocyte; FGF2: basic fibroblast growth factor; FGF4: fibroblast growth factor 4 is a protein-coding gene; FGF9: fibroblast growth factor 9; Gremlin and Noggin: are bone morphogenetic protein antagonists that inhibit BMP signaling; Wnt3A ligand: canonical signaling pathway; NEAA: cell culture supplement is a 100X stock solution containing seven types of nonessential amino acids; oncostatin-M: glycoprotein with an approximate molecular weight 28 kDa, is a pleiotropic cytokine of the IL-6 type; Rspondin-1: is a protein found on chromosome 1 and interacts in humans with Wnt4 in the process of female sexual development; N2 supplement: chemically defined 100X concentrate of Botenstein’s N-2 formulation; Wnt/beta-catenin: protein for signaling; Lgr5: marker for endoderm induction; Wnt: canonical signaling pathway; Y-glucocorticoids: are corticosteroid family hormones involved in the regulation of carbohydrate metabolism, ZRNF3 and RFN43 are two transmembrane E3 ligases that remove Wnt receptors from the surface of stem cells.

## DIFFERENTIATION OF INDUCED PLURIPOTENT STEM CELLS

Once the primary cells have been induced into pluripotent stem cells, it is necessary to differentiate them to obtain the cells that will form the organoid. For this purpose, special factors are used, which are added to the culture medium and are specific for each type of organoid to be obtained. These factors respond to embryonic development. To learn about the processes that occur in nature, particularly in mammals and humans, it is best to imitate them and delve into the genetic, biochemical and physical development that occurs in the evolution of the human being from the fertilization of the egg by the sperm (the first stage from the formation of the zygote), until the outlines of the future organs are established, when the cells are already committed to their irreversible destinies.

## ORGANOID GENERATION

As has been reviewed, three embryonic layers are formed during gastrulation, and each of them derives into different organs, therefore it is essential to first induce the formation of the embryonic layer from which the target organ will be derived. For this purpose, inducing substances are used to stimulate the formation of the corresponding layer.

**Figure 2:**
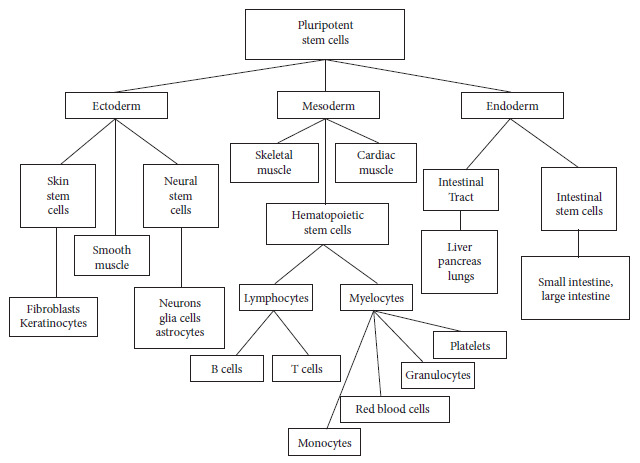
The three induced embryonic layers of pluripotent stem cells and their important differentiations.

Figure 2 shows some of the organs derived from any of the three embryonic layers; the corresponding embryonic layer, stimulated by the specific inducers for its formation, must also receive, in its culture medium, the elements that will stimulate, directly or through signaling pathways, the specific differentiation of the cells that will constitute the expected organ.

Table 1 shows the differentiation elements required for the generation of some organoids such as brain, intestines, liver, kidneys and lungs.

### Activin-A

Activin-A, a dimeric glycoprotein polypeptide, belongs to the transforming growth factor TGF-β family, and regulates various biological functions: signals through the SMAD213 protein cell proliferation, differentiation, apoptosis and metabolism, but mainly stimulates FSH which is a gonadotropin, follicle-stimulating hormone of development, growth, pubertal maturation and reproductive processes in the body. It is synthesized and secreted by the anterior pituitary gland.

### BMP signaling

*Morphogenetic protein*

Endoderm-inducing morphogenetic protein directly regulates or interacts with other pathways to control endoderm specification. Zang and Jianwen ^31^ from Columbia University comment that TGF is a tumor growth factor referred to BMP signaling. The bone morphogenic protein (BMP) pathway is essential for morphogenesis of multiple organs in the digestive tract. BMP signaling regulates gastrointestinal function and disease progression: stem cells, progenitor cells and inflammation. The BMP pathway consists of factors that induce bone and cartilage formation, as well as lung tissues, kidneys, the gastrointestinal tract and associated organs. They modulate many cellular activities, such as proliferation, differentiation and migration in different organs (esophagus, stomach and intestines).

*R-Spondin Family*

R-Spondin1 is an essential growth factor for culture media formulation in the generation of gut organoids. The R-spondin family are factors that enhance Wnt pathway activity in epithelial stem cells, Gremlin-1 or Noggin and BPM pathway differentiation signals. Given the problems of batch-to-batch variation that occur in organoid generation processes, Urbischek *et al*. ^32^, from the University of Cambridge, presented media formulations that allow the derivation and maintenance of organoids from colon, stomach, pancreas, liver, and prostate. These media contain highly pure preparations of R-spondin-1 and Gremlin-1, obtained from bacterial expressions. These are formulations that describe the tasks of growth factor production, quality control and also of the respective culture media.

Recently, Hacker and Ordóñez-Morán ^33^ continued Urbischek’s advances and developed a procedure for large-scale production of recombinant R-spondin-1 and Noggin proteins from human embryonic kidney 293 (HEK293) cells grown in suspension and orbitally agitated in a bioreactor for seven days. The authors report that this system offers high yield and significant efficiency.

In many cases, the formation of the organ is incomplete, as it remains in its fetal state or in a transitory stage and it requires maturation as a final part of the process, therefore, it is necessary to use maturation elements that take the forming organ to its final state of mature organ (Table 1).

## SIGNALING PATHWAYS

Signaling pathways are intercellular communication processes in which a molecule called a ligand binds to another molecule on the surface of a cell, part of a tissue, called a receptor that receives, transmits and activates through a molecule or chain of molecules the processes of cellular, tissue and organoid formation.

Takahashi and Shiraishi ^34^ state that the intestinal epithelium undergoes rapid and continuous self-renewal, cell commitment and differentiation along the crypt-villus axis during the postnatal period. Paneth cells control stem cell and niche signal expressions, such as epidermal growth factor, transforming growth factor α-Wnt3 and ligand-4.

Wnt signaling is controlled by ligands and receptors and also by activators and inhibitors included in R-Spondin-LGR signaling. Intestinal stem cells (ISCs) located at the base of the crypt regulate the balance of their differentiation and self-renewal. Regulated IFN signaling preserves intestinal stem cell potency in control mice ^35^. Interferon regulatory factor-2 (IRF2) negatively affects interferon signaling.

Andrews *et al*. ^36^ reported their experience with the mammalian target of rapamycin (mTOR) signaling pathway that is active in human oRG cells and state that mutations in mTOR pathway genes are associated with various neurodevelopmental disorders and malformations of cortical development. Accordingly, mTOR signaling regulates their morphology and migration.

## DESIGNER MATRICES

Designing and preparing special culture media containing all the elements required for cell maintenance, growth and differentiation, is necessary in order to mimic *in vitro* the physical and biochemical properties of the microenvironment in which cells, as required for their differentiation, develop into a living organism. Matrigel culture medium and type-I collagen are usually used to generate organoids from induced pluripotent stem cells and adult stem cells, but there is concern that in some cases the animal origin of one of the components of the matrigel may affect the organoids formed by giving rise to batch-to-batch variations, diminishing the validity of the experimental work or weakening its conclusions. Therefore, it is recommended that researchers working in these areas should be able to design their own matrices, or at least modify their composition in order to adapt them to the development of the chosen organ and obtain results that meet their expectations, with greater precision and safety.

The matrices prepared in this way are called designer matrices and must consider parenchymal cells, immune cells, vascular cells and neural cells, in order to obtain the expected organoid, one that fits the characteristics of the selected organ. It must also include the elements related to its microenvironment, the extracellular matrix and niche, growth factors, interacting chemical compounds, enzymes and all that emits physical and biochemical signals to achieve growth, tissue morphogenesis and organoids with the defined shape and size, including pro-motor and inhibitory proteins for cell growth, membrane proteins, among others. The matrices can be made of natural or synthetic materials; the latter may allow more precise controls of the cellular microenvironments.

The following parameters give matrices the capacity to modulate formation: stiffness, composition, degradability, geometry, control of the activities of growth factors and capacity to bind these factors. Stiffness is detected by integrins ^37^, and differentiation is promoted by the softest of these (190P.a.). Laminin-511 and laminin-521, from human endodermal cells ^38^, promote differentiation to liver cells.

Experiments on loss of function could identify the components of the extracellular matrix (ECM) and its protein by testing the secretome for its functionality in promoting organoid formation and development. Malta *et al*. ^39^ developed a procedure based on the ECM to study its molecular composition that enable the differentiation of endoderm into liver and pancreatic cells.

Matrices, in general, require a support matrix to maintain the characteristics and functions resembling those of a given organ. Jee *et al*. ^40^ developed a novel matrix based on type-I collagen, Ham’s F12 nutrients and bicarbonate, obtaining organoids from mouse colon and small intestine. The ECM was constituted by collagen and laminin that allow the proliferation of pluripotent stem cells, as well as their differentiation. Collagen is part of the proteins that constitute the ECM of humans and living beings in general and participate in cell migration and division, among other functions.

## MICROCHIPS AND ORGANOIDS

In a recently published article, Naumovska *et al*. ^41^ recognize the contribution of intestinal organoids for drug development. They propose the differentiation of induced pluripotent stem cells to the intestinal phenotype, cultured in a gel on microfluidic chips in which the cells, after forming a tubular structure, lose their stem cell characteristics and acquire those of mature intestinal cells such as enterocytes, Paneth cells and neuroendocrine cells.

Caruso *et al*. ^42^ published a review on the use of microfluidic devices in which they describe the benefits of these technologies and comment on their capacity to quantify chemical species, high sensitivity and analytical speed for the study of drug-organ interactions. The use of microchip electrophoresis (ME) in biological, toxicological and drug studies is highlighted.

## CONCLUSIONS

The predictions about the benefits of the organoid model regarding the biological development of organs and tissues have been widely achieved. This is also true for the onset and progression of diseases, laying the foundations for obtaining drugs and achieving treatments aimed at personalized medicine. In-depth progress regarding signaling pathways is remarkable, as well as all that refers to the elements of differentiation and maturation. The process of organoid generation, in order to further expand its development, must incorporate many more components into the design matrices, in order to progressively improve the similarity of the organoids generated with respect to native organs. To this effect, the natural development process of organs, tissues and even cells (gastrulation) should be further researched by delving into the molecular mechanisms in order to determine the chemical reactions that take place inside cells and tissues, as well as niches and extracellular matrices. Biochemical development is very important at this stage in which the structural identification of components, chemical bonds and also stereochemical structures of ligands can be achieved. Physical aspects will become increasingly important, considering pressures and physical spaces, the mechanical forces applied by the cells to the surrounding tissues, the densities of chemical elements and even the phenomena of attraction and repulsion of electrical charges. Microchips using a microfluidic platform are already available.

These studies emphasize the importance of the variables inherent to the scientific method, causal variables, interfering variables, especially confounding variables, temporal variables and many others, but the search and definitions will always have to be based on very in-depth experimental studies. It is advisable to start the modulation process with the induction of natural cells and reprogramming them, in order to obtain induced pluripotent stem cells, this will show their healthy or diseased characteristics in great detail, which will allow a significant scientific advance. It will also be possible to start directly with natural cells. The generation of organoids associated with CRISPR-CAS9 gene-editing techniques is expected to offer extraordinary and unpredictable results. In addition, the creation of new instruments with unprecedented advances, such as the atomic force microscope, will be able to extend human vision to extraordinary limits and allow us to see molecules and even atoms with greater definition. There is no doubt that these instruments will enable organoids (micro-organs) to be examined with greater depth and precision. This instrumental improvement will improve the identification of elements, which has not yet been achieved with nanotechnology, supporting current work and broadly developing the microchip field.

Technology deserves special mention for its future contributions in the development and application of organoids, which was considered impossible a few years ago. Microbiological studies with bacteria and viruses will continue. So far, studies have focused on the relationship between organoids and *Helicobacter pylori*, and in the case of coronaviruses, particularly with SARS-CoV-2, the cause of the current pandemic. Current major limitations regarding the development of organoids must be considered, especially in order to create the cellular microenvironment required for organ functions, taking into account the diversity and great cellular complexity of the tissues of each organ. The challenges for organoid development, imaging and functional studies, like those regarding contractility, nerve impulse transmission, filtration, etc., should be further evaluated, since organoids require better matrix media. In addition, these challenges demonstrate the lack of available literature regarding neurotransmission and vascular aspects. The progress reached up to this point regarding organoids is the result of the human creative effort that has resulted in sometimes surprising and unexpected conclusions and proposals, which show a remarkable tendency towards scientific improvement.
