# Supplementary material for: Organoids: fundamentals, present and future
Source: Rev Peru Med Exp Salud Publica. 2022 Jun 30;39(2):227–35. doi: 10.17843/rpmesp.2022.392.10203 (PMC11397781; doi:10.17843/rpmesp.2022.392.10203)
Supplement: Supplementary material. — Available in the electronic version of the RPMESP. [file rpmesp-39-02-10203-s001.docx]

*Referencias con aplicaciones de procedimientos para generar organoides.*

*Organoides de cerebro:*

1. Lancaster MA, Knoblich, Organogenesis in a dish: Modeling development and disease using organoid technologies. Science Vol. 345 (6194) 1247125 (2014). doi: 10.1126 / science.1247125
2. Dang J, Tiwari SK, Lichinchi G, et al. Zika Virus Depletes Neural Progenitors in Human Cerebral Organoids through Activation of the Innate Immune Receptor TLR3. *Cell Stem Cell*. 2016; 19(2):258-265. doi:10.1016/j.stem.2016.04.014
3. Dos Reis RS, Sant S, Keeney H, Wagner MCE, Ayyavoo V. Modeling HIV-1 neuropathogenesis using three-dimensional human brain organoids (hBORGs) with HIV-1 infected microglia. Sci Rep. 2020 Sep 16; 10(1):15209. doi: 10.1038/s41598-020-72214-0. PMID: 32938988
4. Irina Rakotoson, Brigitte Delhomme, Philippe Djian, Andreas Deeg, Maia Brunstein, Christian Seebacher, Rainer Martin Oheim.Fast, multicolor 3-D imaging of brain organoids with a new single-objective two-photon virtual light-sheet microscope. Posted November 03, 2018. [doi.org/10.1101/461335](https://doi.org/10.1101/461335)
5. Kathuria, Annie et al. “Transcriptomic Landscape and Functional Characterization of Induced Pluripotent Stem Cell-Derived Cerebral Organoids in Schizophrenia.” *JAMA psychiatry* vol. 77,7 (2020): 745-754. doi:10.1001/jamapsychiatry.2020.0196
6. Lancaster MA, Renner M, Martin CA, et al. Cerebral organoids model human brain development and microcephaly. Nature*;*501(7467): 373-379 (2013). doi:10.1038/nature12517
7. Seo HH, Han HW, Lee SE, Hong SH, Cho SH, Kim SC, Koo SK, Kim JH. Modelling*Toxoplasma gondii*infection in human cerebral organoids. Emerg Microbes Infect. 2020 Dec;9(1):1943-1954. doi: 10.1080/22221751.2020.1812435. PMID: 32820712.
8. Das, Debamitra et al. “Human Forebrain Organoids from Induced Pluripotent Stem Cells: A Novel Approach to Model Repair of Ionizing Radiation-Induced DNA Damage in Human Neurons.” *Radiation research* vol. 194,2 (2020): 191-198. doi:10.1667/RR15567.1

*Organoides Gastrointestinales*

1. Min, Sungjin et al. “Gastrointestinal tract modeling using organoids engineered with cellular and microbiota niches.” Experimental &molecular medicine vol. 52,2 (2020): 227-237. doi:10.1038/s12276-020-0386-0Min
2. S, Kim S, Cho SW. Gastrointestinal tract modeling using organoids engineered with cellular and microbiota niches. ExpMol Med. 2020 Feb;52(2):227-237. doi: 10.1038/s12276-020-0386-0. Epub 2020 Feb 26. PMID: 32103122; PMCID: PMC7062772.
3. Sugimoto, Shinya et al. “Organoid Derivation and Orthotopic Xenotransplantation for Studying Human Intestinal Stem Cell Dynamics.”Methods in molecular biology (Clifton, N.J.) vol. 2171 (2020): 303-320. doi:10.1007/978-1-0716-0747-3_21
4. Cayetano Pleguezuelos-Manzano,Jens Puschhof,Stieneke van den Brink,Veerle Geurts,Joep Beumer,Hans Clevers. “Establishment and Culture of Human Intestinal Organoids Derived from Adult Stem Cells”. Current Protocols in Inmunology. (First published: 17 September 2020); 130(1): e 106. [doi.org/10.1002/cpim.106](https://doi.org/10.1002/cpim.106).
5. Mikhail Nikolaev et al. Homeostatic mini-intestines through scaffold-guided organoid morphogenesis. Nature. (2020 16 setiembre). doi: 1038/s 41586-020-2724-8

*Organoides de Riñones*

1. Beichen Ding, Guoliang Sun, “Organoides renales tridimensionales de células renales completas: generación, optimización y aplicación potencial en nefrotoxicología in vitro”. CLL Cell Transplantation. Publicado por primera vez el 13 de marzo de 2020. [doi.org/10.1177/0963689719897066](https://doi.org/10.1177/0963689719897066)
2. Biao Huang,Zhenqing Liu,Ariel Vonk,Zipeng Zeng,Zhongwei Li, “Epigenetic regulation of kidney progenitor cells”. STEM CELLS Translational Medicine.Volume 9, Issue 6. (First published 12 March 2020). [doi.org/10.1002/sctm.19-0289](https://doi.org/10.1002/sctm.19-0289)

*Organoides: Cancer*

1. Jamo Drost et al. Uso de organoides de células madre humanas modificados con CRISPR para estudiar el origen de las firmas mutacionales en el cáncer. Science. Publicado en línea el 14 de septiembre del 2017. doi: 10.1126 / science.aao3130. PMCID: PMC6038908. EMSID: EMS78533. PMID: 28912133
2. El li 1 2, Weixing Dai et al. Modelado del desarrollo de tumores y metástasis utilizando organoides emparejados derivados de pacientes con metástasis hepáticas de cáncer colorrectal. Journal Hematol Oncol. 2020 3 de septiembre; 13 (1): 119. doi:10.1186 / s13045-020-00957-4. PMID: 32883331 PMCID: PMC7650218

*Organoides: Covid-19*

1. Anand Ramani et al. “El SARS-CoV-2 se dirige a las neuronas de los organoides del cerebro humano en 3D”. EMBO Journal 2020. EMBO J (2020) 39: e106230 [doi.org/10.15252/embj.2020106230](https://doi.org/10.15252/embj.2020106230)
2. Kelvin Kai-Wang Para et al. Reinfección por COVID-19 por una cepa filogenéticamente distinta del coronavirus-2 del SARS confirmada por secuenciación del genoma completo. 2020 25 de agosto; ciaa1275. doi: 10.1093 / cid. PMID: 32840608 PMCID: PMC7499500
3. Rajasekaran Mahalingam et al. “Análisis de secuenciación de ARN unicelular de los receptores de entrada del SARS-CoV-2 en organoides humanos”. Cellular Physiology. Publicado por primera vez: 17 de septiembre de 2020. [doi.org/10.1002/jcp.30054](https://doi.org/10.1002/jcp.30054).
4. Sang Ah Yi et al. "Infección de organoides cerebrales y neuronas corticales 2D con pseudovirus SARS-CoV-2”. Virus 2020, 12 (9), 1004. [doi.org/10.3390/v12091004](https://doi.org/10.3390/v12091004)
5. Trevisan M, Riccetti S, Sinigaglia A, Barzon L. SARS-CoV-2 Infection and Disease Modelling Using Stem Cell Technology and Organoids. Int J Mol Sci. 2021 Feb 26; 22(5):2356. doi:10.3390/ijms22052356. PMID: 33652988
6. Dickson I. Organoids demonstrate gut infection by SARS-CoV-2. Nat Rev Gastroenterol Hepatol. 2020 Jul; 17(7):383. doi:10.1038/s41575-020-0317-5.PMID: 32427981
7. Tiwari SK, Wang S, Smith D, Carlin AF, Rana TM. Revealing Tissue-Specific SARS-CoV-2 Infection and Host Responses using Human Stem Cell-Derived Lung and Cerebral Organoids..Stem Cell Reports. 2021 Mar 9; 16(3):437-445. doi: 10.1016/j.stemcr.2021.02.005. Epub 2021 Feb 12.PMID: 33631122
8. Mahalingam R, Dharmalingam P, Santhanam A, Kotla S, Davuluri G, Karmouty-Quintana H, Ashrith G, Thandavarayan RA. Single-cell RNA sequencing analysis of SARS-CoV-2 entry receptors in human organoids..J Cell Physiol. 2021 Apr; 236(4):2950-2958. doi:10.1002/jcp.30054. Epub 2020 Sep 17.PMID: 32944935
9. Jurado-Gómez A, Giraldez MD. Nueva enfermedad por coronavirus-2019 y el tracto gastrointestinal: lecciones aprendidas de los organoides humanos. Gastroenterología. 2020 diciembre; 159 (6): 2245-2247. doi:10.1053/j.gastro.2020.09.039. Epub 2020 1 de octubre.PMID: 33010249
10. De Oliveira M, De Sibio MT, Costa FAS, Sakalem ME. Los organoides de las vías respiratorias y los alvéolos como valiosas herramientas de investigación en COVID-19. ACS Biomater Sci Ing. 9 de agosto de 2021; 7(8):3487-3502. doi:10.1021/acsbiomaterials.1c00306. Epub 2021 21 de julio.PMID: 34288642
11. Liuliu Yang, Yuling Han, Benjamin E. Nilsson-Payant, Vikas Gupta, PengfeiWang, Xiaohua Duan, Tang Xuming, jiajun zhu, Zeping Zhao, Fabrice Jaffré, tuo zhang, Tae Wan Kim, Oliver Harschnitz, david redmond, Sean Houghton, Chengyang Liu, Alí Naji, Gabriele Ciceri, Sudha Guttikonda, Yaron Bram, Duc-Huy T Nguyen, Michele Cioffi, Vasuretha Chandar, Margarita un Hoagland, Yaoxing Huang, Jenny Xiang, hui wang, david lyden, Alain Borczuk, HuanhuanJoyce Chen, Lorenz Studer, Sartén Fong Cheng, David D Ho dieciséis, Benjamin R tenOever, Roberto E Schwartz, Shuibing Chen. Una plataforma basada en células madre pluripotentes humanas para estudiar el tropismo del SARS-CoV-2 y modelar la infección por virus en células humanas y organoides. Stem Cell. 2 de julio de 2020; 27 (1): 125-136.e7. doi:10.1016/j.stem.2020.06.015. Epub 2020 19 de junio.
