# Supplementary material for: Organoids: fundamentals, present and future
Source: Rev Peru Med Exp Salud Publica. 2022 Jun 30;39(2):227–35. doi: 10.17843/rpmesp.2022.392.10203 (PMC11397781; doi:10.17843/rpmesp.2022.392.10203)
Supplement: Translation

# Translation: Organoides: fundamentos, presente y futuro

**Authors:** José Amiel-Pérez, José Amiel-Sáenz, María Amiel-Torrelio

**Affiliations:** 1 Instituto de Medicina Regenerativa, Universidad Científica del Sur, Lima, Perú.; 2 Georgia Southern University Statesboro, Georgia, United States.

**Keywords:** Células madre pluripotentes inducidas, Gastrulación, Matrices de diseño, Cultivos tridimensionales, Diferenciación celular, Vías de señalización, Maduración celular, Microchips, Cepas embrionarias, Factores de crecimiento

## Abstract

Los organoides son estructuras miniaturizadas, generadas principalmente a partir de células madre pluripotentes inducidas, que se cultivan en el laboratorio conservando sus características innatas o adquiridas. Tienen el potencial de reproducir procesos de desarrollo biológico, modelar procesos patológicos que permitirán el descubrimiento de nuevos fármacos y propicien la medicina regenerativa. Sin embargo, estas experiencias requieren perfeccionamiento constante porque pueden haberse realizado variaciones en la constitución de estos órganos. Por ello, el presente artículo tiene como objetivo revisar la información actualizada sobre organoides y sus procesos experimentales básicos y recientes, empezando por la gastrulación, para tratar de imitar, en lo posible, la formación de las tres capas: ectodermo, mesodermo y endodermo, incluyendo los factores que intervienen en la inducción, diferenciación y maduración en la generación de estos organoides. Asimismo, el diseño y preparación de medios de cultivo altamente especializados que permitan obtener el órgano seleccionado con la mayor precisión y seguridad. Se realizó una búsqueda de artículos originales y de revisión publicados en PubMed, Nature y Science. Los artículos se seleccionaron por sus resúmenes y por su texto completo. Las conclusiones de este articulo destacan las ventajas futuras en el uso y aplicaciones de los organoides.

## INTRODUCCIÓN

Los organoides son estructuras celulares miniaturizadas derivadas de tejidos primarios o de células madre diferenciadas y autoorganizadas tridimensionalmente (3D) *in vitro *que muestran arquitectura física y funcionalidad orgánica similares a las de los órganos-objetivo: el cerebro, el hígado, riñones, estómago, intestinos, y muchos más, que permiten el modelado, tanto de órganos normales, como de los enfermos ^1^ ^-^ ^5^ y tienen potencial para reproducir procesos de desarrollo biológico ^6^ ^,^ ^7^. Estos organoides harán posible modelar procesos patológicos y evidenciar aspectos desconocidos que permitirán el descubrimiento de nuevos fármacos, verificar la calidad de las drogas, efectuar ensayos toxicológicos ^8^ ^-^ ^10^ así como tratamientos médicos más precisos conducentes a propiciar la medicina personalizada y regenerativa. La construcción de organoides que puedan reemplazar funcionalmente (aunque sea en parte) la función de algún órgano dañado se proyecta como una estrategia de la medicina regenerativa ^11^. El objetivo del presente artículo es revisar la información actualizada sobre organoides y sus procesos experimentales básicos y recientes.

## ESTRATEGIA DE BUSQUEDA Y SELECCIÓN

Se realizó una búsqueda de artículos originales y de revisión publicados en PubMed, Nature y Science. Se limitó a artículos en inglés y español. Se usaron las palabras: organoides, gastrulación, cultivos celulares, matrices de diseño, vías de señalización, microchips y COVID-19. Los artículos se identificaron por los títulos, aproximadamente entre 250-300, solo los referidos a los temas elegidos. Se seleccionaron los resúmenes de 102 artículos, que luego de una evaluación del texto completo se seleccionaron 42 que se muestran en este artículo y posteriormente se ampliaron a 70 al incluir aplicaciones específicas de las técnicas para obtener organoides correspondientes a diversos órganos: cerebro, intestinos, riñones y aplicaciones para COVID-19. Estas 28 referencias restantes están disponibles en el material suplementario.

## AUTOORGANIZACIÓN

En 1907 Wilson publicó su trabajo experimental «Un nuevo método por el cual las esponjas pueden ser criadas artificialmente», el que mostraba cómo las células de las esponjas divididas en células individuales y separadas unas de otras tenían la capacidad de autoorganizarse reconstituyendo así el organismo vivo ^12^.

Posteriormente, aparecieron publicaciones referidas a experiencias en las que se obtenía in vitro la reconstitución completa de órganos por reagregación de células. Sin embargo, los avances más notables en el área del desarrollo para la obtención de organoides se han logrado a partir del perfeccionamiento de cultivos tridimensionales y técnicas que derivaron del uso de células madre pluripotentes inducidas.

## CULTIVOS CELULARES

Las células madre reproducidas en capas bidimensionales crecen en superficies planas y no representan a las células originales, pierden su fenotipo y no son adecuadas para generar organoides, debido a que no muestran interacciones célula-célula o célula-matriz ni imitan las funciones de las células, tampoco las vías de señalización. En cambio, los cultivos tridimensionales (3D) exhiben una representación más precisa del ambiente natural del organismo. Por eso, son muy importantes para la formación de organoides.

El medio en el que se cultivan es importante para el crecimiento, desarrollo y posterior diferenciación de las células madre. En los sistemas *in vivo*, son importantes los conceptos de microambiente y nicho (constituido por las capas externas de las células inmediatas al microambiente), en donde destaca la matriz extracelular y el conjunto de interacciones específicas entre las células de esa región. Para los sistemas *in vitro* en donde se desea generar organoides, es clave el uso de matrices que imiten dicha interacción.

Esta generación de organoides requiere como elemento base a los tejidos primarios, que son los que conforman el organismo animal y humano, o a las células madre, en particular las células madre pluripotentes inducidas (iPSC). Estas iPSC son las más apropiadas para su derivación a organoides y no tienen impedimentos éticos ^13^, como sí los tienen las células embrionarias (ESC) que impiden el desarrollo del embrión imposibilitando una vida humana.

## CÉLULAS MADRE PLURIPOTENTES INDUCIDAS

Las células madre pluripotentes inducidas tienen la cualidad de ser autorrenovables ^14^ y de proporcionar las señales físicas y bioquímicas que se necesitan para crear un organoide humano. Las señales físicas son fundamentales para constituir la arquitectura morfológica del organoide, aportando apoyo para la conexión entre célula y célula, y el crecimiento celular; también participan en esta tarea los componentes de los medios de cultivo, entre ellos destacan el colágeno y la laminina. Las señales bioquímicas son responsables de la diferenciación, proliferación y autorrenovación en el proceso de generación de organoides, complementados con la Wnt (Wingless y Int-1), gastrina, ácido retinoico, inhibidores de HDAC, EGF. Se requieren para emular el microambiente celular, conocerlo mejor, a su microbiota y, particularmente, su vascularización ^15^ ^,^ ^16^.

Hace algunos años se identificaron células madre adultas de la médula ósea y el tejido graso que derivan en células de más de una capa germinal ^17^. El año 1998, Thomson *et al*. ^18^ ^) ^cultivaron *in vitro* células pluripotentes tomadas del blastocisto. Utilizaron medios de cultivo de enriquecimiento que estimulan su crecimiento. Así, las células madre cultivadas vuelven a modificar su característica asimétrica y se convierten en células madre simétricas al generar dos células iguales que se multiplican y expanden rápidamente. El estudio de Thomson *et al*. fue pionero en conseguir células madre a partir de la masa interna del blastocisto y es muy importante porque fue una experiencia que antecede a los estudios posteriores de Yamanaka ^19^. En el 2007, Takahashi *et al*. ^20^ lograron obtener, con el mismo método, células madre pluripotentes inducidas humanas.

Por la versatilidad de estas células, los científicos especialistas la utilizaron para la obtención de organoides sanos o enfermos, preservando sus rasgos genéticos mutados o inalterados. Para lograrlo había que transformar previamente las células a procesar en células madre pluripotentes inducidas; después era necesario tomar las muestras apropiadas y aplicarles las técnicas actualizadas propuestas por Yamanaka. Una vez inducidas deberán ser expandidas y diferenciadas para recapitular el órgano seleccionado.

## GASTRULACIÓN

La gastrulación es un proceso que tiene lugar después de la formación de la blástula y forma el disco embrionario como consecuencia de la migración de poblaciones celulares que se dirigen al epiblasto originando la formación de las tres capas embrionarias.

Durante el desarrollo natural del embrión, y después de la formación de la mórula, se constituye el blastocisto, una estructura transitoria embrionaria que aparece entre 4-6 días después de la fecundación, previa a su implantación en el endometrio en donde se forman de 70-100 células llamadas blastocistos que conforman la masa celular interna (MCI), células que son pluripotentes y se encuentran en el interior de la cavidad central o blastocele, formando el trofectodermo (TE) que constituirá la placenta y las membranas amnióticas.

Al finalizar la segunda semana se inicia la gastrulación, un proceso que tiene lugar después de la formación de la blástula y forma el disco embrionario como consecuencia de la migración de poblaciones celulares que se dirigen al epiblasto, originando la formación de las tres capas embrionarias: ectodermo, mesodermo y endodermo, proceso que conduce a las especificaciones celulares para la generación natural de los distintos órganos. Termina a fines de la cuarta semana.

Las células del disco embrionario constituidas por una masa aplanada se organizan en dos láminas, una que será parte de la región dorsal del futuro embrión denominada epiblasto y la otra el hipoblasto, en la parte inferior. Ambas capas conforman el blastodermo que es el origen de todas las células y tejidos embrionarios y también de parte de los extraembrionarios.

La formación de nuevos organismos necesita de la creación de variadas láminas capaces de formar todos los elementos constitutivos de las tres capas embrionarias: ectodermo, mesodermo y endodermo ^21^, inicialmente constituidas solo por dos, por lo que se hace imperativa la formación de una tercera capa, el mesodermo, que se suma a las dos anteriores, epiblasto e hipoblasto, aportados por el disco embrionario laminar, el embrión trilaminar ^22^.

Para el efecto, algunas células epiblásticas migran, una parte de ellas se introducirá entre las capas del epiblasto y el hipoblasto con dirección al blastocele y la otra se incorporará al grupo de células que constituyen la capa del hipoblasto. Esta migración da lugar a la formación de la línea primitiva, que es el aspecto morfológico que presentan las células cuando están ingresando a través de ella para formar el mesodermo, proceso migratorio que se denomina invaginación y genera una estructura de aspecto alargado de nombre notocorda, la que luego inducirá a la formación del tubo neural, la placa neural y los cuerpos vertebrales ^23^.

La invaginación origina una disminución del tamaño de la cavidad blastocélica, especificada por factores de crecimiento de fibroblastos-8 (FGF-8) que disminuyen la concentración de cadherina-E, proteínas que mantienen unidas a las células epiblásticas facilitando así la migración celular. Esta disminución permite la formación de una nueva cavidad, el gastrocelo, futuro intestino.

Las células epiblásticas que no migraron y quedaron, constituirán el ectodermo; la capa que recién ha resultado de la invaginación es el mesodermo, y la nueva constituida por el conjunto de las células del hipoblasto más las epiblásticas, conforman el endodermo.

López *et al*. ^24^ destacan el hecho de que las tres láminas embrionarias se van desarrollando simultáneamente y forman distintos órganos con características propias y específicas para cada uno de ellos, con importantes interacciones moleculares, celulares y tisulares entre sí. También mencionan que muchos de los datos obtenidos en embriología y los más relevantes en los procesos de gastrulación devienen del estudio de aves (de gallinas) pero que, sin duda, son trasladables a la especie humana. A continuación, se resume el desarrollo de cada capa.

### Ectodermo

Constituye la capa externa del embrión y es parte del saco amniótico que rodea al embrión y contiene al líquido amniótico. La parte central, el ectodermo neural, formará el sistema nervioso y el ectodermo no-neural será la epidermis.

### Mesodermo

Forma un gran número de órganos, capa intermedia que resulta de la internización de la blástula de las células de la capa superior. La notocorda, su elemento más importante, tendrá una participación fundamental en los procesos de inducción neural. Se ha demostrado que cuando se extirpa la notocorda no tiene lugar la diferenciación del ectodermo neural. El mesodermo propiamente dicho se divide en tres sectores: el mesodermo paraxial, el intermedio y el lateral. El mesodermo paraxial conforma los pares de somnites. Las células que componen los somnites se diferencian a tres tipos de estructuras celulares: miotomo, dermotomo y esclerotomo. 

### Endodermo

Es la capa embrionaria interna, la que con mínimos cambios sigue el proceso de incurvación embrionaria desde la boca hasta el ano, formando el tubo digestivo y particularmente a la mucosa digestiva (Figura 1).

**Figura 1:** Formación de las tres capas embrionarias en el proceso natural de gastrulación.

El sistema cardiovascular y el sistema nervioso se constituyen en la tercera semana de gestación ^25^. El sistema circulatorio es la primera unidad en funcionar y el corazón lo hace tan pronto se le requiera para cubrir las necesidades de oxígeno y nutrientes ^26^.

## DIFERENCIACIÓN EN LA NATURALEZA

La expresión genética específica es la responsable de la codificación y producción de las proteínas que señalizan y transmiten información intercelular y son las generadoras de la información intercelular, mecanismo en el que participan moléculas inductoras, represoras y procedimientos de control. Participan en esta regulación génica factores de transcripción que modulan la expresión de ciertos genes y vías de señalización que, utilizando un procedimiento de señales, establece una comunicación intercelular por el que una molécula denominada ligando se une a la superficie de un receptor celular que transmite y activa a través de una molécula, o una cadena de moléculas (vía de señalización) los procesos intracelulares de la formación celular, tisular y organoidal. Por ejemplo, en el caso de la neurodiferenciación es importante el rol del ligando BMP4, que es la proteína morfogenética ósea codificada por el gen *Bmp4*. Cuando no hay actividad *Bmp4* el ectodermo dorsal forma tejido neural por defecto; entonces, la notocorda produce los compuestos anti-BMP nogina, folistatina y cordina, inhibidores de la proteína. Las interacciones moleculares estimulan a las células ectodérmicas para su transformación a tejido neural. La subdivisión del sistema nervioso central es regulada por los genes *Otx2, Gbx2 y Hox *que regulan la distribución regional. La expresión del gen *Otx2* es importante para el desarrollo de las regiones del prosencéfalo y mesencéfalo. Los genes *Gbx2 y Hox *estarían implicados en la generación del romboencéfalo. La regionalización del tubo neural se hace evidente en la región del cerebro posterior con el gen *Hox*.

La expresión primera corresponde a la evolución y diferenciación del corazón ^27^ ^,^ ^28^ se inicia con el factor de transcripción Nkx-2.5, resultante de la inducción de los factores de crecimiento fibroblástico Fgf (*Fibroblast-growth-factors*) y Bmp (*Bone-morphogenetic-protein*)^ (^ ^29^, proteínas morfogenéticas de hueso que se expresan en el endodermo ubicado junto al mesodermo precardíaco y compromete a estas células como vías de diferenciación cardiacas ^30^. En la Tabla 1 se muestran los principales factores de inducción y diferenciación celular en los diferentes tejidos.

**Tabla1. Factores:** de inducción, de diferenciación y maduración para la generación de organoides.

Organoides	Elementos de diferenciación
Cerebro	Suplemento N2, NEAA, heparina para la inducción neural. Insulina, 2-mercaptoetanol para la diferenciación. Vitamina A, ácido retinoico para maduración.
Intestinos	Activina-A, BMP4, señalización Wnt /beta-catenina, marcador Lgr5 para la inducción del endodermo. FGF4, ligandoWnt3A. Rspondin-1 para la diferenciación. Gremlin y Noggin, EGF, FGF4, Wnt, afamina, ZRNF3, RFN43 (ubiquitinasas) para maduración.
Hígado	Activina-A para la inducción del endodermo. BMP4, FGF2, factor de crecimiento de hepatocitos para la diferenciación. Oncostatina-M para maduración.
Riñones	Wnt, inhibidor de GSK3α para la inducción del mesodermo intermedio. FGF9 para la diferenciación
Pulmones	Activina-A para la inducción del endodermo. Wnt, BMP, FGF, cAMP. Y-glucocorticoides para la diferenciación.

Activina-A: proteína de estructura dimérica; afamina: glicoproteína que transporta vitamina E; BMP: proteína morfogenética ósea; BMP4: proteína morfogenética ósea 4 es un gen codificador de proteínas; cAMP: monofosfato de adenosina cíclico, segundo mensajero utilizado para la inducción de señales intracelulares; EGF: el factor de crecimiento epidérmico es una proteína que estimula el crecimiento y la diferenciación celular combinándose con su receptor EGFR; FGF: el factor de crecimiento fibroblástico-23 es una proteína sintetizada en el osteocito; FGF2: factor de crecimiento de fibroblastos básico; FGF4: factor de crecimiento de fibroblastos 4 es un gen codificador de proteínas; FGF9: factor de crecimiento de fibroblastos 9; Gremlin y Noggin: son antagonistas de la proteína morfogenética ósea que inhiben la señalización de BMP; ligando Wnt3A: vía de señalización canónica; NEAA: suplemento de cultivo celular es una solución madre 100x que contiene siete tipos de aminoácidos no esenciales; oncostatina-M: glicoproteína con un peso molecular aproximado 28 kDa, es una citoquina pleiotrópica del tipo IL-6; Rspondin-1: es una proteína que se encuentra en el cromosoma 1 interactúa en humanos con Wnt4 en el proceso de desarrollo sexual femenino; suplemento N2: concentrado 100X químicamente definido de la formulación N-2 de Botenstein; Wnt/beta-catenina: proteína para señalización; Lgr5: marcador para la inducción del endodermo; Wnt: vía de señalización canónica; Y-glucocorticoides: son hormonas de la familia de los corticosteroides que participan en la regulación del metabolismo de carbohidratos, ZRNF3 y RFN43 son dos ligasas E3 transmembrana que eliminan los receptores Wnt de la superficie de las células madre.

## DIFERENCIACIÓN DE CÉLULAS MADRE PLURIPOTENTES INDUCIDAS

Una vez inducidas las células primarias a células madre pluripotentes es necesario diferenciarlas para obtener las células que conformarán el organoide. Para ello se emplean factores especiales que se agregan al medio de cultivo y son específicos para cada tipo de organoide por obtener. Estos factores responden al desarrollo embrionario. Para conocer los procesos que ocurren en la naturaleza, en particular en los mamíferos y el hombre, lo mejor es imitarlos y profundizar en el desarrollo genético, bioquímico y físico que se da en la evolución del ser humano desde la fertilización del óvulo por el espermatozoide (la primera etapa desde la formación del cigoto), hasta que se establecen los esbozos de los futuros órganos, cuando las células ya están comprometidas a sus destinos irreversibles. 

## GENERACIÓN DE ORGANOIDES

Como se ha revisado, en la gastrulación se forman tres capas embrionarias y cada una de ellas deriva a distintos órganos, por ello es imprescindible inducir primero la formación de la capa embrionaria de la cual se derivará el órgano objetivo. Para esto se utilizan las sustancias inductoras que estimulan la formación de la capa correspondiente.

En la Figura 2 se observan algunos de los órganos que derivan de cualquiera de las tres capas embrionarias; la capa embrionaria correspondiente, estimulada por los inductores específicos para su formación, debe recibir, además en su medio de cultivo, los elementos que van a estimular, directamente o a través de las vías de señalización, la diferenciación específica de las células que constituirán el órgano esperado.

En la Tabla 1 se observan los elementos de diferenciación requeridos para la generación de algunos organoides como los de cerebro, intestinos, hígado, riñones y pulmones.

**Figura 2:** Las tres capas embrionarias inducidas de las células madre pluripotentes y sus diferenciaciones importantes.

### Activina-A

La activina-A, polipéptido de la glucoproteína dimérica, pertenece a la familia del factor de crecimiento transformante TGF-β, y regula diversas funciones biológicas, señala a través de la proteína SMAD213 la proliferación celular, la diferenciación, la apoptosis y el metabolismo, pero principalmente estimula a la FSH que es una gonadotropina, hormona foliculoestimulante del desarrollo, el crecimiento, la maduración puberal y los procesos reproductivos del cuerpo. Es sintetizada y secretada por la glándula pituitaria anterior.

### Señalización BMP

*Proteína morfogenética*

La proteína morfogenética inductora del endodermo regula directamente o interactúa con otras vías para controlar la especificación del endodermo. Zang y Jianwen ^31^ ^) ^de la Universidad de Columbia comentan que TGF es un factor de crecimiento tumoral referido a la señalización BMP. La vía de la proteína morfogénica ósea (BMP) es esencial para la morfogénesis de múltiples órganos en el aparato digestivo. La señalización BMP regula la función gastrointestinal y la progresión de la enfermedad: células madre, células progenitoras e inflamación. La vía BMP está constituida por factores que inducen la formación de huesos y cartílago, además de tejidos pulmonares, riñones, el tracto gastrointestinal y órganos asociados. Modulan muchas actividades celulares, como la proliferación, diferenciación y migración en diferentes órganos (esófago, estómago e intestinos).

*Familia de la R Spondinas*

R-Spondin1 es un factor esencial de crecimiento para la formulación de medios de cultivo en la generación de organoides de intestino; la familia de las R-espondinas son factores que potencian la actividad de la vía Wnt en las células madre epiteliales, Gremlin-1 o Noggin y las señales de diferenciación de la vía BPM. Ante los problemas de variación lote a lote que ocurren en los procesos de generación de organoides, Urbischek *et al*. ^32^, de la Universidad de Cambridge, presentaron formulaciones de medios que permiten derivar y mantener organoides de colon, estómago, páncreas, hígado y próstata. Estos medios contienen preparaciones altamente puras de R-espondin-1 y Gremlin-1, obtenidas de expresiones bacterianas. Son formulaciones que describen las tareas de producción de factores de crecimiento, control de calidad y también de los medios de cultivo respectivos.

Recientemente Hacker y Ordóñez-Morán ^33^ continuando los avances de Urbischek, desarrollaron un procedimiento de producción a gran escala de proteínas recombinantes R-espondin-1 y Noggin a partir de células de riñón embrionario humano 293 (HEK293) cultivadas en suspensión y agitadas orbitalmente en un biorreactor durante siete días. Los autores comunican que este sistema ofrece un alto rendimiento y eficiencia significativa.

En muchos casos la formación del órgano no se reproduce completamente, permanece en su estado fetal o en una etapa transitoria, entonces requiere madurarse como parte final del proceso, por lo cual, es necesario utilizar elementos de maduración que lleven al órgano en formación a su estado final de órgano maduro (Tabla 1).

## VÍAS DE SEÑALIZACIÓN

Las vías de señalización son procesos intercelulares de comunicación en los que una molécula denominada ligando se une a otra que se encuentra en la superficie de una célula, parte de un tejido, denominada receptor que recibe, transmite y activa a través de una molécula o cadena de moléculas los procesos de formación celular, tisular y organoidal.

Takahashi y Shiraishi ^34^ indican que el epitelio intestinal experimenta una autorrenovación rápida y continua, un compromiso y una diferenciación celular a lo largo del eje cripta-vellocidad durante el período posnatal. Las células Paneth controlan a las células madre y a las expresiones de señales de nicho, tales como el factor de crecimiento epidérmico, el factor de crecimiento transformante α-Wnt3 y el ligando-4.

La señalización de Wnt es controlada por ligandos y receptores y también por activadores e inhibidores incluidos en la señalización R-Spondin-LGR. Las células madre intestinales (ISC) que se ubican en la base de la cripta regulan el equilibrio de su diferenciación y autorrenovación. La señalización regulada de IFN preserva la potencia de las células madre intestinales en ratones utilizados como control ^35^. El factor regulador de interferón-2 (IRF2) afecta negativamente la señalización del interferón.

Andrews *et al.* ^(^ ^36^ comunican sus experiencias con la vía de señalización de rapamicina en mamíferos (mTOR) que está activa en las células oRG humanas y afirman que las mutaciones en los genes de la vía mTOR están asociadas con diversos trastornos del neurodesarrollo y malformaciones del desarrollo cortical. En consecuencia, la señalización mTOR regula su morfología y migración. 

## MATRICES DE DISEÑO

Para imitar *in vitro las* propiedades físicas y bioquímicas del microambiente en el que desarrollan las células en un organismo vivo, como se requiere para su diferenciación, es necesario diseñar y preparar medios de cultivo especiales que contengan todos los elementos requeridos para el mantenimiento, crecimiento y diferenciación celular. Usualmente se utilizan el medio de cultivo matrigel y el colágeno tipo-I, particularmente para generar organoides a partir de células madre pluripotentes inducidas y de células madre adultas, pero preocupa que en algunos casos el origen animal de uno de los componentes del matrigel pueda afectar a los organoides que se formen al dar lugar a variaciones lote a lote, disminuyendo la validez del trabajo experimental o debilitando sus conclusiones. Por eso es recomendable que los investigadores que trabajen en estas áreas puedan diseñar sus propias matrices, o cuando menos modificar su composición a fin de adecuarlas al desarrollo del órgano que se ha elegido y obtener resultados que se ajusten a lo esperado, con mayor precisión y seguridad.

Las matrices así preparadas se denominan matrices de diseño y exigen tener en cuenta a las células parenquimatosas, las células inmunes, las células vasculares y las neurales, para lograr el organoide que se espera, uno que se ajuste a las características del órgano seleccionado. Debe, además, incluir todo lo relacionado con su microambiente, la matriz extracelular y el nicho, los factores de crecimiento, los compuestos químicos que interactúan, las enzimas y todo lo que emita señales físicas y bioquímicas que permitan lograr el crecimiento, la morfogénesis de tejidos y los organoides con la forma y tamaño definidos, e incluyan proteínas promotoras e inhibidoras del crecimiento celular, proteínas de membranas, entre otras. Las matrices pueden fabricarse con materiales naturales o sintéticos; estos últimos tal vez pudieran permitir controles más precisos de los microambientes celulares.

Los parámetros que otorgan a las matrices capacidad para modular la formación son: rigidez, composición, degradabilidad, geometría, control de las actividades de los factores de crecimiento y capacidad de unir a estos factores. De todos ellos destaca el primero, la rigidez, que es detectada por las integrinas ^37^ en donde las más suaves (190P.a.) promueven la diferenciación. La laminina-511 y la laminina-521, a partir de células endodérmicas humanas ^38^, promueven la diferenciación a células hepáticas.

Si se realizaran experimentos con pérdidas de una función determinada se podría determinar los componentes de la matriz extracelular (ECM) y de su proteína ensayando con el secretoma la funcionalidad en la promoción de la formación y desarrollo de organoides. Malta *et al*. ^39^ desarrollaron un procedimiento basado en la ECM para estudiar las moléculas que la constituyen y hacen posible la diferenciación del endodermo a células hepáticas y pancreáticas.

Las matrices, en general, requieren de una matriz de soporte para continuar con las características y funciones similares a las de un órgano determinado. Jee *et al*. ^40^ desarrollaron una matriz nueva basada en colágeno tipo-I, nutrientes Ham´s F12 y bicarbonato, obteniendo organoides de colon y del intestino delgado de ratón. La ECM estaba constituida por colágeno y laminina que permiten la proliferación de las células madre pluripotentes, además de su diferenciación. El colágeno es parte de las proteínas que constituyen la ECM de los humanos, los seres vivos en general y participan en la migración y división celular, entre otras funciones.

## MICROCHIPS Y ORGANOIDES

Naumovska *et al*. ^41^ en un artículo recientemente publicado reconocen la contribución de los organoides intestinales para el desarrollo de fármacos. Plantean la diferenciación de células madre pluripotentes inducidas al fenotipo intestinal, cultivados en un gel en chips microfluídicos en los que las células, luego de formar una estructura tubular, pierden sus características de células madre y adquieren las de células intestinales maduras como la de enterocitos, células de Paneth y neuroendocrinas.

Caruso *et al*. ^42^ publicaron un trabajo de revisión sobre el uso de dispositivos microfluídicos en el que describen los beneficios de estas tecnologías y comentan su capacidad para cuantificar especies químicas, alta sensibilidad y rapidez analítica para el estudio de las interacciones fármaco-órgano. Se destaca la utilización de la electroforesis de microchips (EM) en estudios biológicos, toxicológicos y de fármacos.

## CONCLUSIONES

Las previsiones sobre la bondad del modelo de organoides referidas al desarrollo biológico de órganos y tejidos se han cumplido ampliamente. Lo mismo en cuanto al inicio y progreso de las enfermedades, sentando las bases para la obtención de fármacos y el logro de tratamientos conducentes a la medicina personalizada. El avance en profundidad sobre el tema de las vías de señalización es notable, así como todo lo que se refiere a los elementos de diferenciación y maduración. El proceso de generación de organoides para continuar ampliando su desarrollo, debe incorporar muchos más ingredientes a las matrices de diseño, de manera tal que se perfeccione progresivamente la similitud de los organoides generados con respecto a los órganos nativos. Para este efecto deberá investigarse más el proceso natural de desarrollo de órganos, tejidos e incluso células (gastrulación) penetrando a los mecanismos moleculares de modo que se determinen incluso las reacciones químicas que tienen lugar al interior de células y tejidos, así como nichos y matrices extracelulares. El desarrollo bioquímico será muy importante en esta etapa en la que podrá llegarse a la identificación estructural de los componentes, enlaces químicos y también estructuras estereoquímicas de los ligandos. Los aspectos físicos serán cada vez más importantes, considerándose para ello las presiones y los espacios físicos, las fuerzas mecánicas aplicadas por las células a los tejidos que las rodean, las densidades de los elementos químicos y hasta los fenómenos de atracción y repulsión de las cargas eléctricas. Ya existen microchips que utilizan una plataforma microfluídica.

En estos estudios se destaca la importancia de las variables propias del método científico, las variables causales, las interferentes, especialmente las de confusión, temporales y muchas más, pero su búsqueda y definición habrá de ocurrir siempre con estudios experimentales de mucha profundidad. Para comenzar el proceso de modulación es recomendable iniciarlo con la inducción de las células naturales, reprogramándolas, para obtener células madre pluripotentes inducidas, porque ellas evidenciarán sus características sanas o enfermas muy minuciosamente, lo que permitirá un avance científico significativo. También podrá iniciarse directamente con las células naturales. Se ha previsto que la generación de organoides asociados a las técnicas de edición genética CRISPR-CAS9 ofrecerán resultados extraordinarios e imprevisibles. Además, la creación de nuevos instrumentos con avances inimaginables, como puede ocurrir con el microscopio de fuerzas atómicas que podrá ampliar la visión humana hasta conducirnos a límites extraordinarios y permitirán ver con mayor definición las moléculas y aun los átomos. No hay duda de que estos instrumentos harán posible examinar los organoides (microórganos) con mayor profundidad y precisión. Este perfeccionamiento instrumental impulsará la identificación de elementos que aún no se ha logrado con la nanotecnología, apoyando trabajos actuales y desarrollando ampliamente el área de los microchips.

La tecnología merece una mención especial por sus aportes futuros en el desarrollo y aplicación de organoides, considerados imposibles hace pocos años. Continuarán los estudios microbiológicos con bacterias y virus. Hasta ahora se ha trabajado con insistencia organoides en su relación con el *Helicobacter pylori*, y en el caso de los coronavirus, particularmente lo será con el SARS-CoV-2, causante de la pandemia que azota actualmente a la humanidad. Debe considerarse las grandes limitaciones que se tiene actualmente en el desarrollo de los organoides, sobre todo para generar el microambiente celular requerido para las funciones del órgano, tomando en cuenta la diversidad y gran complejidad celular de los tejidos de cada órgano. Los desafíos en el desarrollo de organoides, estudios de imágenes y funcionales: contractilidad, transmisión del impulso nervioso, filtración, etc. deberán evaluarse, ya que requieren mejores medios matriciales y exhiben significativas ausencias referidas particularmente a los aspectos vasculares y la neurotransmisión. Toda esta evolución lograda a la fecha con los organoides es el resultado del esfuerzo creativo humano que ha llegado a conclusiones y propuestas a veces sorpresivas e inesperadas, fruto de la creatividad humana, desarrollo que exhibe una notable tendencia al perfeccionamiento científico.
